# Facile fabrication of large-grain CH_3_NH_3_PbI_3−*x*_Br_*x*_ films for high-efficiency solar cells via CH_3_NH_3_Br-selective Ostwald ripening

**DOI:** 10.1038/ncomms12305

**Published:** 2016-08-01

**Authors:** Mengjin Yang, Taiyang Zhang, Philip Schulz, Zhen Li, Ge Li, Dong Hoe Kim, Nanjie Guo, Joseph J. Berry, Kai Zhu, Yixin Zhao

**Affiliations:** 1 Chemistry and Nanoscience Center, National Renewable Energy Laboratory, 15013 Denver West Parkway, Golden, Colorado 80401, USA; 2 School of Environmental Science and Engineering, Shanghai Jiao Tong University, 800 Dongchuan Road, Shanghai 200240, China

## Abstract

Organometallic halide perovskite solar cells (PSCs) have shown great promise as a low-cost, high-efficiency photovoltaic technology. Structural and electro-optical properties of the perovskite absorber layer are most critical to device operation characteristics. Here we present a facile fabrication of high-efficiency PSCs based on compact, large-grain, pinhole-free CH_3_NH_3_PbI_3−x_Br_x_ (MAPbI_3−*x*_Br_*x*_) thin films with high reproducibility. A simple methylammonium bromide (MABr) treatment via spin-coating with a proper MABr concentration converts MAPbI_3_ thin films with different initial film qualities (for example, grain size and pinholes) to high-quality MAPbI_3−*x*_Br_*x*_ thin films following an Ostwald ripening process, which is strongly affected by MABr concentration and is ineffective when replacing MABr with methylammonium iodide. A higher MABr concentration enhances I–Br anion exchange reaction, yielding poorer device performance. This MABr-selective Ostwald ripening process improves cell efficiency but also enhances device stability and thus represents a simple, promising strategy for further improving PSC performance with higher reproducibility and reliability.

## Abstract (web summary)

Organolead halide perovskite optoelectronic devices require high material quality. Here, Yang *et al*. show that methylammonium lead iodide films can undergo Ostwald ripening and become mixed-halide films using methylammonium bromide treatment. This processing increases both the device efficiency and stability.

## Graphical abstract

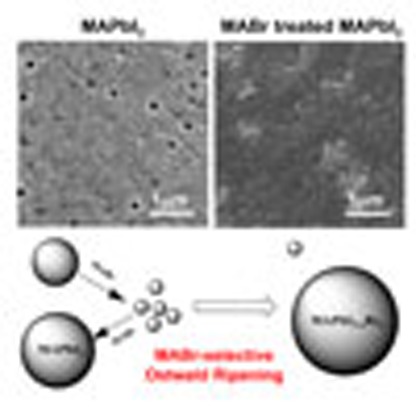

Organometallic halides perovskite (for example, CH_3_NH_3_PbI_3_ or MAPbI_3_) solar cells have demonstrated exceptional, unparalleled progress in solar cell performance from 3.8% in 2009 to a certified 22.1% by now^1^,^2^,^3^,^4^,^5^,^6^,^7^,^8^,^9^,^10^,^11^,^12^,^13^,^14^,^15^,^16^,^17^,^18^. Crystallization-engineered one-step methods without using an additive have become most popular for the solution processing of perovskite films. Ultrasmooth perovskite films are usually prepared by the solvent-engineering, fast-crystallization process, and/or Lewis base adduct approaches^8^,^19^,^20^,^21^. In all these one-step methods, high-quality perovskite or perovskite intermediate films were achieved by timely application of antisolvent such as toluene or chlorobenzene to the precursor solution (for example, a mixture of PbI_2_ and methylammonium iodide (MAI) in dimethylformamide or γ-butyrolactone/dimethylsulfoxide (DMSO)) during the spin-coating process under room temperature, which generally results in a fast nucleation and crystallization process. Because of the general complex processes involved in perovskite formation^22^, the processing for making high-quality perovskite films has become an art owing to the very narrow time window for properly adding the antisolvent; this could potentially be responsible for some reproducibility problems, especially the large variations among different groups. The fast-crystallization process of perovskite usually forms nanocrystals with broad size distribution and could introduce defects due to the rapid crystallization process^19^,^23^. The uncertainty of size distribution of nanocrystals and densities of defects can, in part, explain the wide distribution of device performance from different groups or researchers. Moreover, the material purity of perovskite precursors and the atmosphere of perovskite film deposition will greatly affect the final perovskite film quality as well^24^,^25^,^26^. Although the performance of perovskite solar cells (PSCs) fabricated with these one-step techniques has reached ∼18%–20% by several groups, it is normally ∼15%–16% for most laboratories because of the aforementioned reasons. Some post-treatment techniques, such as the solvent-annealing process, have been proposed to recrystallize the small-size crystals into large perovskite grains with reduced pinhole formation; however, these methods also require some skilled control techniques, which are difficult to master by most laboratories^14^,^27^. Therefore, it is necessary to develop a facile one-step solution method for obtaining higher-quality perovskite films without involving delicate processing controls (or more skill tolerance). Ideally, some mediocre perovskite films (from solvent-engineering methods) can be easily converted into high-quality perovskite films (large grain size and free of pinholes) for high-performance PSCs. Ostwald ripening is one of the most popular and simplest ways to refine the small crystals into large ones via a thermodynamically spontaneous process^28^,^29^. However, there is no report of adapting the Ostwald ripening process to obtain large-grain-size lead halide perovskite films for high-efficiency PSCs.

Here we demonstrate that by a facile post treatment of the methylammonium bromide (MABr) solution-induced Ostwald ripening process, the MAPbI_3_ thin films (with relatively small grains, broad size distribution and pinholes at times) can be converted into high-quality MAPbI_3−*x*_Br_*x*_ perovskite thin films with large grain size and no pinholes. This MABr-refining treatment is simply carried out by spin-coating the existing MAPbI_3_ thin film with a 2 mg ml^−1^ MABr isopropanol (IPA) solution. This novel MABr-induced Ostwald ripening treatment can be applied to either a high-quality or low-quality MAPbI_3_ film for forming higher-quality pinhole-free perovskite thin films with large grain sizes. With this facile MABr-induced Ostwald ripening process, we could obtain 19.12% efficiency MAPbI_3−*x*_Br_*x*_ PSCs, which contrasts significantly to the efficiency level of ∼14–16% for the MAPbI_3_ films without this MABr treatment. Furthermore, the MAPbI_3−*x*_Br_*x*_ perovskite film with a small amount Br infiltration (∼1%) has shown much-improved stability at elevated temperature (∼100 °C) in comparison with the MAPbI_3_ films without the MABr treatment.

## Results

### Structural and electro-optical characterization

**Figure 1:**
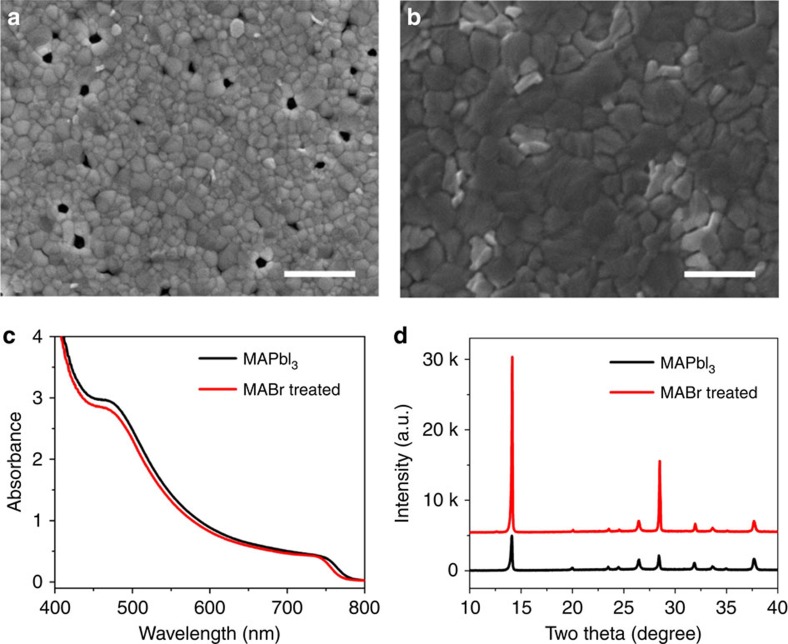
Impact of MABr treatment on the structural and electro-optical properties of MAPbI_3_ thin film. Top view of the SEM images of (**a**) MAPbI_3_ and (**b**) MABr-treated MAPbI_3_ films. Scale bars, 1 μm. Comparison of (**c**) UV-vis absorption spectra and (**d**) X-ray diffraction patterns of MAPbI_3_ films with and without MABr treatment.

Planar perovskite thin films prepared by solvent-engineering or fast-crystallization method often look smooth and compact. However, when the antisolvent is not applied accurately (that is, an unsuccessful application), significant pinholes are found within the films as often noted from scanning electron microscopy (SEM) images. The PSCs fabricated with mediocre or low-quality perovskite films with low crystallinity and pinholes usually exhibit relatively low (for example, less than 15%) device efficiencies. Figure 1 compares the effect of MABr treatment on the SEM images of film morphology, ultraviolet-visible (UV-vis) absorption spectra, X-ray diffraction patterns and photoluminescence (PL) spectra of the perovskite thin films. For the results shown in Fig. 1, a 2 mg ml^−1^ MABr solution was used to conduct the MABr treatment. Figure 1a shows that the grains for low-quality MAPbI_3_ films are generally smaller than 500 nm and a noticable number of pinholes can be observed. Surprisingly, the morphology of these low-quality MAPbI_3_ films turns into compact perovskite films with larger micron size after the MABr treatment. Figure 1c shows that the MABr treatment causes a slight blue shift (less than 5 nm) in the absorption, which could be attributed to the Br inclusion. No significant difference in the absorption intensity is observed between the pristine MAPbI_3_ and MABr-treated films. It is interesting that the crystallinity of the perovskite film appears to be enhanced significantly with the MABr treatment. Figure 1d shows that the X-ray diffraction peak intensity of the MAPbI_3_ film increases by about five times without any obvious peak shift after the 2 mg ml^−1^ MABr treatment. The X-ray diffraction results indicate that the 2 mg ml^−1^ MABr treatment has significantly enhanced the crystallinity of the average perovskite film but with little Br incorporation for forming MAPbBr_3_ or I–Br mixed perovskite. The signficant change in the crystallinity after the MABr treatment is consistent with the grain-morphology changes as shown in the SEM images. It is also worth noting that the MABr-treated film exhibits a stronger PL intensity and longer decay time (Supplementary Fig. 1) than the MAPbI_3_ sample, which is consistent with the overall improvments in the MAPbI_3−*x*_Br_*x*_ film quality that may also lead to a potential passivation effect with the MABr treatment.

### Typical device characteristics

**Figure 2:**
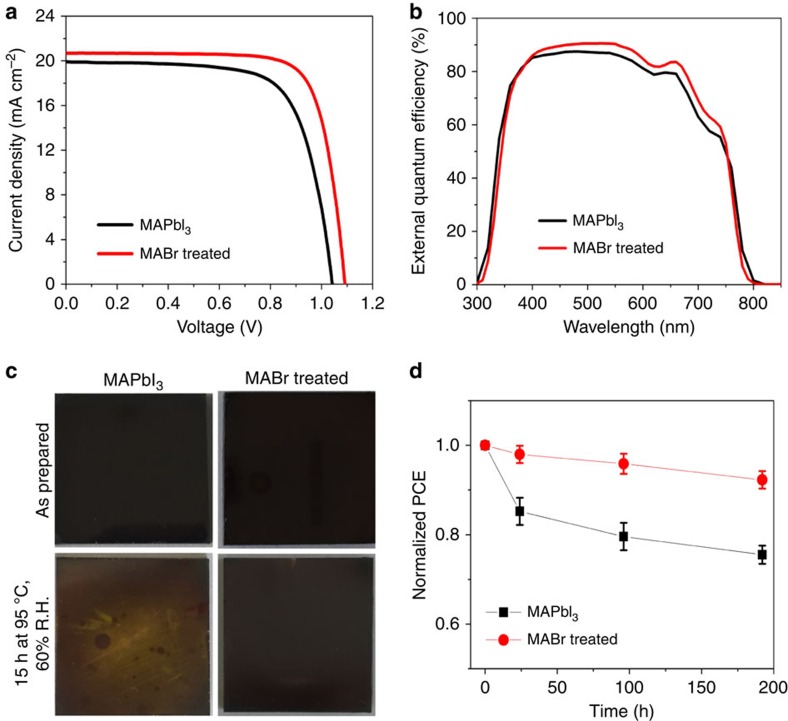
Device characteristics and stability comparison. Comparison of (**a**) *J*–*V* characteristics and (**b**) EQE spectra for planar perovskite solar cells based on MAPbI_3_ thin films with and without MABr treatment. (**c**) Comparison of the photographs of as-prepared MAPbI_3_ thin films (with and without MABr treatment) versus those subjected to 15 h exposure to 60% relative humidity (R.H.) at 95 °C. (**d**) Device stability comparison of perovskite solar cells based on MAPbI_3_ thin films with and without the MABr treatment. The error bars represent the s.d.'s from eight devices.

Figure 2a shows the typical photocurrent density–voltage (*J–V*) curves of the MAPbI_3_- and MAPbI_3−*x*_Br_*x*_-based solar cells. It is noteworthy that for these devices the initial MAPbI_3_ films have mediocre (relatively poor) film quality as shown in Fig. 1a. However, we achieve a substantial performance improvement with MABr treatment. The MAPbI_3_-based PSC shows ∼14.65% power conversion efficiency (PCE) with a short-circuit photocurrent density (*J*_sc_) of 19.93 mA cm^−2^, open-circuit voltage (*V*_oc_) of 1.042 V and fill factor (FF) of 0.705. With the 2 mg ml^−1^ MABr treatment, the device performance for MAPbI_3−*x*_Br_*x*_-based PSC increases to 17.38% with a *J*_sc_ of 20.68 mA cm^−2^, *V*_oc_ of 1.092 V and FF of 0.770. On the basis of the analysis of 8–12 devices, the average efficiency (with s.d.) improves from 14.76±0.54% to 17.37±0.01% with the MABr treatment. The detailed statistic results of photovoltaic parameters of these devices are shown in Table 1. The improved device performance with all photovoltaic parameters is consistent with the improved structural and electro-optical properties (for example, higher crystallinity, larger grain size and fewer surface defects, and improved PL properties) of MAPbI_3−*x*_Br_*x*_ films in comparison with the pristine MAPbI_3_ films. Figure 2b compares the effect of MABr treatment on the external quantum efficiency (EQE) spectra. The integrated *J*_sc_ values are 19.42 and 20.07 mA cm^−2^ for the MAPbI_3_- and MABr-treated devices, respectively. The slight spectral shift near the EQE onset around 800 nm for the MAPbI_3−*x*_Br_*x*_ PSC is consistent with the observed blue shift in the absorption spectra (Fig. 1c). It is interesting that MABr-treated samples show higher EQE values across a wide spectral range despite their similar absorbance values in this spectral range. This indicates that the MABr treatment also improves the charge-collection processes. In addition to the higher PV performance in efficiency, the MABr-treated MAPbI_3−*x*_Br_*x*_ perovskite films also exhibit an enhanced stability. The yellowing shown in Fig. 2c is a clear indication of decomposition of the MAPbI_3_ film. In contrast, the MAPbI_3−*x*_Br_*x*_ film shows an improved thermal stability in air at a humidity level of ∼60% and temperature of 95 °C. Comparison of the stability of non-encapsulated PSCs based on MAPbI_3_- and MABr-treated MAPbI_3_ films is given in Fig. 2d. About 8–12 individual cells were measured for each type of device to check the statistics of device performance. The MAPbI_3−*x*_Br_*x*_ PSC is much more stable than the ones based on pure MAPbI_3_ films. This stability improvment can be attributed to Br inclusion and large crystal grain size^30^,^31^.

### MABr concentration effect

**Figure 3:**
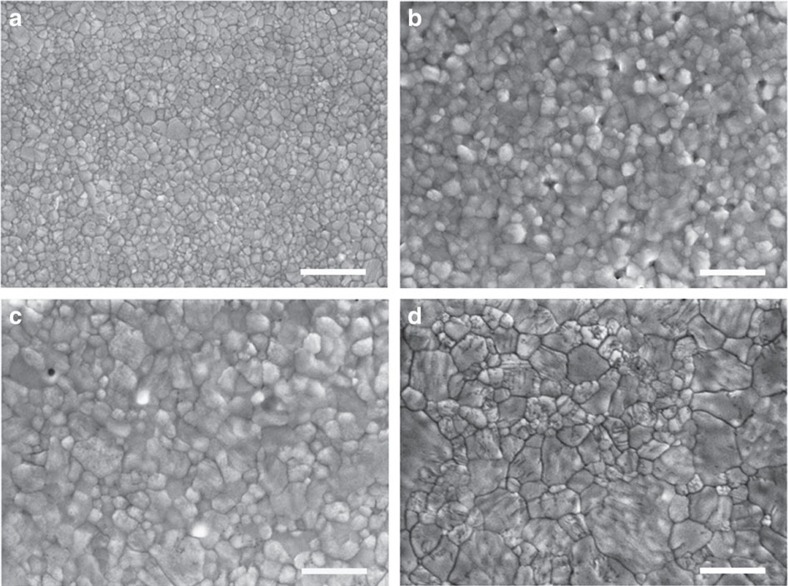
MABr concentration effect on pinhole-free MAPbI_3_ thin films. Top view of SEM images of (**a**) a pinhole-free high-quality MAPbI_3_ thin film and those treated with (**b**) 8 mg ml^−1^, (**c**) 4 mg ml^−1^, and (**d**) 2 mg ml^−1^ MABr solutions. Scale bars, 1 μm.

To further explore the importance of the MABr solution and its concentration for this observed film and device improvement, we examined the effect of varying MABr solution concentration on the properties of the final treated MAPbI_3−*x*_Br_*x*_ films. The average or low-quality MAPbI_3_ films prepared with solvent-engineering can vary significantly in different batches and/or different areas of the same sample; therefore, we adopted the high-quality MAPbI_3_ films cut from a large-area sample for more reliable material property investigation. Figure 3a–d shows the SEM images of a high-quality MAPbI_3_ film (following a successful solvent-engineering process) and those high-quality MAPbI_3_ films treated with different MABr concentrations (from 2 to 8 mg ml^−1^). This high-quality MAPbI_3_ film is compact and pinhole-free with crystallite sizes ranging from 200 to 500 nm. With an 8 mg ml^−1^ MABr treatment, the crystal size of MAPbI_3−*x*_Br_*x*_ becomes larger than the MAPbI_3_ film; however, there is clear evidence of pinhole formation and the surface also becomes rougher, which is similar to the films prepared with the spin-coating-based sequential deposition method. When the MABr concentration is reduced to 4 mg ml^−1^, the MAPbI_3−*x*_Br_*x*_ film becomes less coarse with larger crystallite sizes than the MAPbI_3−*x*_Br_*x*_ film treated with 8 mg ml^−1^ MABr solution. With further reduction of the MABr concentration to 2 mg ml^−1^, the MAPbI_3−*x*_Br_*x*_ film becomes compact with micron-sized (up to 1–2 μm) grains packed with some small perovskite nanocrystals. Supplementary Fig. 2 shows the statistical analysis of the grain-size distribution for these perovskite films. With MABr treatment, the size distribution of the perovskite nanocrystals becomes broader, along with a clear growth of the average particle size. It is worth noting that such dramatic morphology change is similar to those obtained from a low-quality MAPbI_3_ film (Fig. 1a,b), suggesting that this simple post-growth treatment with a low-concentration MABr solution can effectively reconstruct perovskite thin films such that the initial structural defects from a low-quality perovskite thin film can be significantly healed. Further reducing the MABr concentration to 1 mg ml^−1^ does not seem to promote significant grain growth as does the 2 mg ml^−1^ solution (Supplementary Fig. 3c), suggesting that a proper MABr concentration is required.

**Figure 4:**
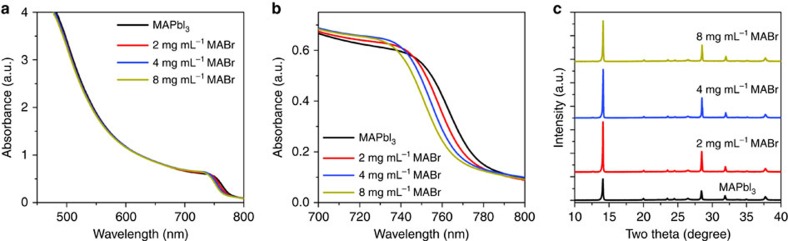
MABr concentration effect on absorption and structure of MAPbI_3_ thin films. (**a**) UV-vis absorption spectra with (**b**) a zoom-in view near the absorption edge, and (**c**) X-ray diffraction patterns of the high-quality MAPbI_3_ thin films without and with MABr treatment at different concentrations, as indicated.

With increasing MABr concentration, the UV-vis absorption shows a systematic blue shift of the absorption edge for the MABr-treated MAPbI_3−*x*_Br_*x*_ (Fig. 4a,b), suggesting a higher amount of Br incorporation. When the high-quality MAPbI_3_ film is treated with an 8 mg ml^−1^ MABr solution, the UV-vis absorption spectrum of the resulting film exhibits a blue shift of the absorption edge of ∼12 nm but no obvious change in the absorbance. It is well known that the MAPbI_3_ can be converted into MAPbI_3−*x*_Br_*x*_ or MAPbBr_3_ via halide exchange reaction once soaked in MABr alcohol solution^32^,^33^. The shift in the absorption edge with increasing MABr concentration is a clear indication of Br inclusion into MAPbI_3_ to form the mixed-halide MAPbI_3−*x*_Br_*x*_, which is consistent with the X-ray diffraction results (Fig. 4c). The (110) peak location increases very slightly but noticeably from ∼14.10° to 14.14° with 8 mg ml^−1^ MABr treatment. However, the peak intensity reaches a maximum at 2 mg ml^−1^ MABr followed by a continuous decrease with higher MABr concentration, which is likely caused by the deterioration of the grain morphology (Fig. 3a–d).

**Figure 5:**
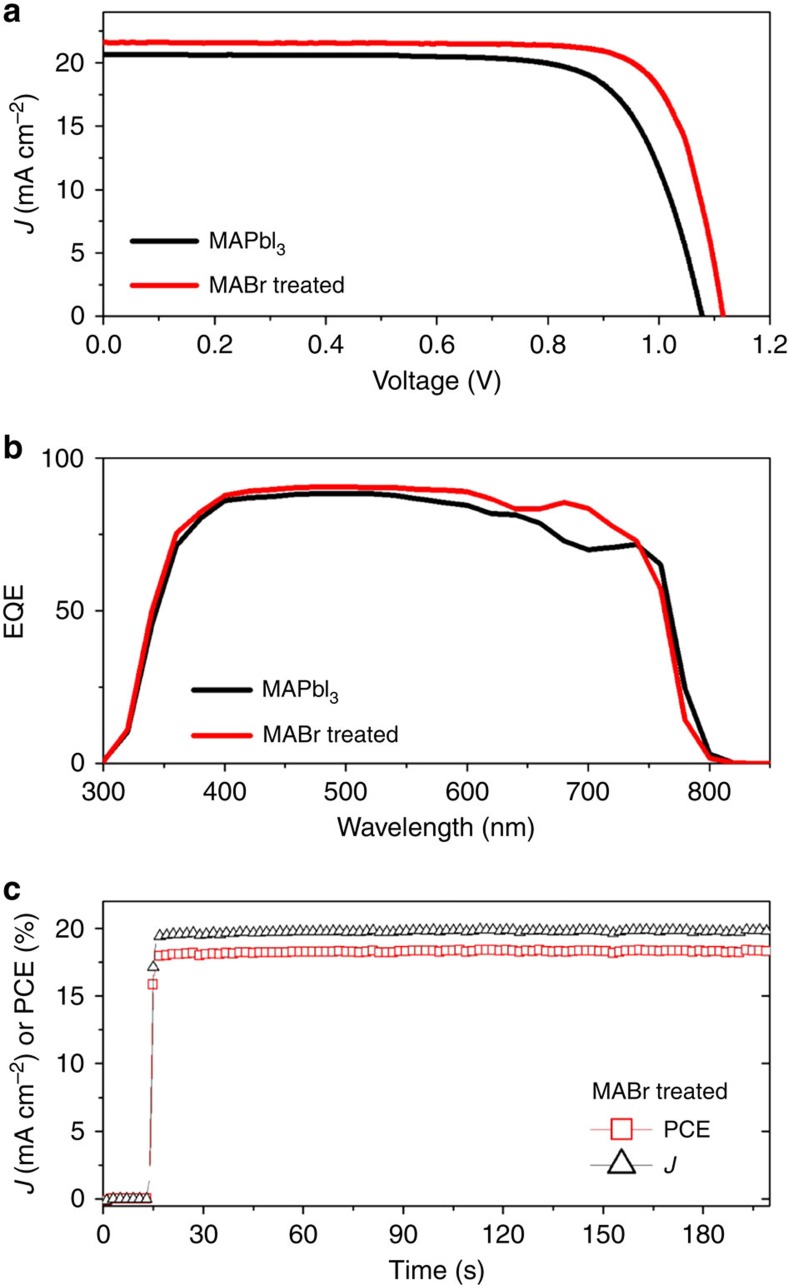
Device characteristics based on high-quality pinhole-free MAPbI_3_ thin films with and without MABr treatment. (**a**) *J–V* curves, (**b**) EQE spectra and (**c**) stabilized photocurrent density and power conversion efficiency biased near the maximum power point. MABr (2 mg ml^−1^) solution was used for the MABr treatment.

Although the compositional change of the MAPbI_3_ film caused by the mild 2 mg ml^−1^ MABr treatment is very minimum as reflected by the small absorption edge shift (∼5 nm) and almost identical X-ray diffraction peak positions (shifted by ∼0.01°–0.02°), the morphology change and X-ray diffraction peak intensity increase are very dramatic, and more importantly, such changes have very little dependence on initial MAPbI_3_ film quality. The very small compositional change is consistent with the X-ray photoelectron spectroscopy (XPS) measurement, which suggests a small amount (∼1%) of Br incorporation in the surface layer of 2 mg ml^−1^ MABr-treated perovskite film. Figure 5a shows the *J–V* curves for PSCs based on the high-quality MAPbI_3_ films with and without the 2 mg ml^−1^ MABr treatment. The pristine MAPbI_3_-based PSC displays a best PCE of 16.63% with *J*_sc_ of 20.67 mA cm^−2^, *V*_oc_ of 1.08 V and FF of 0.746. Such a performance level is also consistent with previous reports using the standard solvent-engineering or fast-crystallization processing. With the MABr treatment, the device efficiency reaches 19.12% with *J*_sc_ of 21.60 mA cm^−2^, *V*_oc_ of 1.12 V and FF of 0.793. The improvement of device parameters is very similar to the devices based on average/low-quality perovskite film. Figure 5b compares the EQE spectra of the two devices shown in Fig. 5a. The integrated current densities are 20.26 and 21.22 mA cm^−2^ for the MAPbI_3_ and MABr-treated devices, respectively, matching well with their corresponding *J*_sc_ values from the *J–V* curves. It is interesting to note that the EQE response with MABr treatment shows improvement in the red region, which might be associated with the potential improvement on the charge extraction at the back side of the device. Because there is still a noticable (and expected) hysteresis behaviour for this compact-TiO_2_-based planar PSC (Supplementary Fig. 4), a stabilized power output was measured over time near the maximum power output point to verify the performance of the PSC. Figure 5c shows that a 18.3% PCE was steadily recorded over several minutes, which matches the *J–V* curve reasonably well and further attests to the effectiveness of the simple MABr treatment to improving device performance.

In contrast to the much-improved device performance with 2 mg ml^−1^ MABr treatment, the PSCs based on 8 and 4 mg ml^−1^-treated MAPbI_3−*x*_Br_*x*_ films exhibit lower device performance, especially the one with 8 mg ml^−1^ MABr treatment. The statistical results of the MABr concentration effect on the photovoltaic parameters of devices are shown in Table 2. For comparison, their typical stabilized current and power outputs were also measured over time near the maximum power output point (Supplementary Fig. 5). This concentration dependence is consistent with the structure/morphology changes discussed in connection with Fig. 3 along with the change in stoichiometry. These results suggest that the simple Br incorporation could not significantly increase PSC performance by only forming MAPbI_3−*x*_Br_*x*_ perovskite films with relatively high Br concentration without affecting the perovskite film/structure properties.

## Discussion

**Figure 6:**
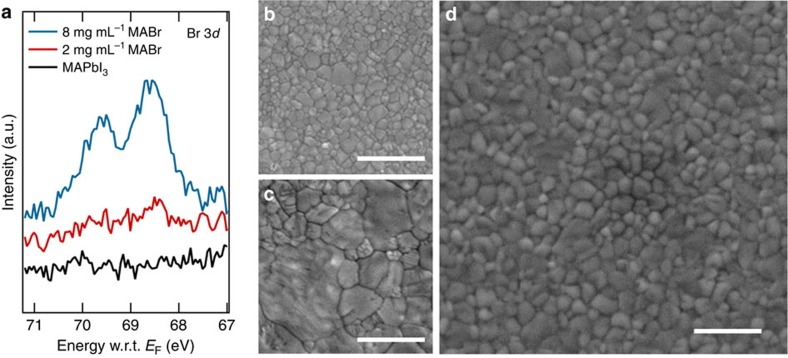
Effect of MABr treatment on XPS core-level analysis and film morphology. (**a**) XPS spectra of the Br 3*d* core-level region for MAPbI_3_ thin films and those treated with low (2 mg ml^−1^) and high (8 mg ml^−1^) concentration MABr solutions. Top-view SEM images of (**b**) a high-quality MAPbI_3_ thin film and those treated with 2 mg ml^−1^ (**c**) MABr and (**d**) MAI solutions. Scale bars, 1 μm.

Figure 6a shows the XPS measurements of the MAPbI_3_ films after 2 and 8 mg ml^−1^ MABr treatment. In addition to the strong I 3*d* signal for all samples (Supplementary Fig. 6), there is a clear but weak Br 3*d* signal for the 2 mg ml^−1^ MABr-treated films. XPS measurements indicate a very low concentration of less than 1% for bromine in the halide content in the surface layer. Such low Br concentration is consistent with the X-ray diffraction results and UV-vis spectra of the MABr-treated MAPbI_3−*x*_Br_*x*_ film (Fig. 4). In contrast, the 8 mg ml^−1^ MABr-treated MAPbI_3−*x*_Br_*x*_ has almost eight times higher Br concentration than the 2 mg ml^−1^ MABr-treated sample.

The above-mentioned results have clearly demonstrated that our MABr treatment with proper concentration is an effective approach to convert an average MAPbI_3_ perovskite film into a high-quality MAPbI_3−*x*_Br_*x*_ film with larger crystal size and higher crystalline order. Such obvious crystal grain-size growth observed with the 2 mg ml^−1^ MABr treatment is consistent with a typical Ostwald ripening phenomenon. It seems that the MABr solution with low concentration such as 2 mg ml^−1^ could induce the Ostwald ripening to recrystallize the small-grain-sized MAPbI_3_ into perovskite films with much enlarged grain size. In contrast, the high-concentration MABr treatment could only induce a regular Br/I halide exchange reaction in the MAPbI_3_ film without significantly affecting the grain growth via distinct Ostwald ripening. The most interesting finding for this Ostwald ripening-based post-growth treatment process is largely invariant to the initial MAPbI_3_ film quality with a similar treatment result (Figs 1 and 3). In the following, we propose a plausible mechanism for understanding the film reconstruction process with MABr treatment.

In the standard solvent-engineering approach, the crystal growth is accelerated by the ultrafast formation of oversaturated precursor solution for the crystal nucleation and growth. With this strategy, a compact perovskite film can be easily formed, but the timing of adding antisolvent to the precursor solution during spin-coating is very critical, as previously demonstrated in ref. 19 because the perovskite precursor film undergoes several crystallization stages during the spin-coating process. Thus, the entire antisolvent-engineering process is kinetically controlled, which makes it highly sensitive to the process conditions. As a result, the atomspheric condition (for example, temperature, humidity and pressure) would introduce uncertainties. Although it has been one of most successful methods adopted by most research groups, there is also a wide spread of device performance among different laboratories, as illustrated by our low-quality and high-quality perovskite films when the experiments are not carried out with a strict control of experimental conditions (which is common for most laboratories). With the Ostwald ripening process, all these different-sized nanocrystals formed at different stages could then grow into similar large crystal size. If we consider the entire solvent engineering plus MABr treatment process as demonstrated in our study, the whole process of fabricating high-quality MAPbI_3−*x*_Br_*x*_ can be divided into two steps. The first step is the formation of MAPbI_3_ precursor nanocrystals by solvent engineering to form a relatively compact thin film. The second step is the conversion of MAPbI_3_ precursor nanocrystals into high-quality MAPbI_3−*x*_Br_*x*_ perovskite film with large crystal size via the MABr-induced Ostwald ripening, which is the key step for determining the final perovskite film properties. A schematic illustration of our MABr treatment procedure and mechanism for the MABr-selective Ostwald ripening process for the perovskite crystal growth is shown in Supplementary Fig. 7. It is worth noting that there is no strict requirement about the time delay between these two steps. Regardless of the initial film quality from the first step, the final crystal sizes obtained from the second step are mainly determined by the thermodynamic Ostwald ripening process, which is less sensitive to experimental operation. This is consistent with the observation that our MABr treatment can convert both the high-quality and low-quality MAPbI_3_ perovskite films into a similar final MAPbI_3−*x*_Br_*x*_ thin film. The Ostwald ripening process normally involves two coupled steps. The first step is the dissolution of small-sized crystals because of the higher surface energy, whereas the second step is the formation of large-sized crystals with lower surface energy. We have further found that simple annealing alone does not cause much growth of perovskite crystals (Supplementary Fig. 8). This is similar to the result by using IPA treatment in the absence of MABr (Supplementary Fig. 3b). It is clear that the dissolution of small crystals is necessary in order to have Ostwald-type ripening, which is possible because the lead halide perovskite MAPbI_3_ can be dissolved in IPA via a back-extracting process of MAI from MAPbI_3_ (refs 34, 35). It has been proposed that perovskite crystal grains could be grown larger through surface-sensitive dissolution/recrystallization processes in the hot casting method and Cl/I mixed-halide perovskite preparation via the two-step method^36^,^37^. These proposed growth mechanisms are all driven by the surface free energy, which is similar to our proposed Ostwald-type ripening. The growth of perovskite via intercalation of the MAI/MABr process depends on the MAI/MABr concentration^38^. In the high-concentration (for example, 8 mg ml^−1^) MABr solution, the dissolution of small-grain-size MAPbI_3_ could be inhibited by the intercalation of MABr or the I/Br cation exchange reaction, which could account for less morphology change or grain-size growth found with the high-concentration MABr treatment. In the low-concentration (for example, 2 mg ml^−1^) MABr treatment, the small-sized MAPbI_3_ crystals can be quickly dissolved and regrown into larger crystals via the Ostwald-type ripening process. We have further examined the effect of directly dipping MAPbI_3_ film in the 2 mg ml^−1^ MABr solution with various durations (from 5 s to 5 min) on the optical absorption (Supplementary Fig. 9) and morphologies (Supplementary Fig. 10) of the resulting perovskite films. The results are similar to that obtained from the high-concentration MABr spin-coating treatment with evidences pointing to (1) substantial Br incorporation, (2) no drastic enhanced grain size and (3) significant pinhole formation, suggesting that the I/Br exchange reaction dominates during the dipping treatment.

To further test whether the observed perovskite reconstruction is not simply resulting from excess of organic salt, we examined the effect of using a similar MAI treatment on the morphology changes of MAPbI_3_ film. Figure 6d shows the SEM image of a high-quality MAPbI_3_ film after post-growth treatment with 2 mg ml^−1^ MAI solution. For comparison, the SEM images of the pristine MAPbI_3_ film and the MABr-treated MAPbI_3−*x*_Br_*x*_ film are shown in Fig. 6b,c, respectively. The grain size increases slightly with MAI treatment; however, the degree of grain growth is much more limited in comparison with the MABr treatment. The best PSC based on MAI treatment shows a PCE of ∼17.11% (Supplementary Fig. 11), which is significantly lower than the performance of a PSC based on the MABr treatment. When a 2.8 mg ml^−1^ MAI solution—the same molar concentration as 2 mg ml^−1^ MABr solution—was used to treat the perovskite films, the perovskite crystallites did not show the same degree of grain size growth as with a 2 mg ml^−1^ MABr solution treatment (Supplementary Fig. 3e). Also noteworthy is that treating the film with the MACl solution (1.2 mg ml^−1^) not only did not show much improvement in crystal grain size, but in addition it also created significant pinholes (Supplementary Fig. 3f). The presence of many pinholes in the MACl-treated sample suggested that MACl may promote the dissolution of perovskites without contributing substantially to the Ostwald ripening process. Typical device characteristics with statistics of MAI-, MACl- and IPA-treated solar cells are compared with MABr-treated cells in Supplementary Fig. 12 and Supplementary Table 1. These results suggest that, besides the MABr concentration, using MABr itself (rather than MAI or MACl) is also important for the Ostwald-type ripening process. The reason for this could be that the Br-terminated perovskite (resulting from the Br/I ion-exchange reaction) may exhibit a lower surface energy, which would induce a more significant Ostwald ripening effect than MAPbI_3_.

In summary, a CH_3_NH_3_Br-selective Ostwald ripening process was developed to effectively fabricate high-quality MAPbI_3−*x*_Br_*x*_ films with micron-sized grains. The regular solvent-engineering approaches usually yield MAPbI_3_ films with different quality (grain size and pinhole formation) due to the rapid reaction under narrow and strict experimental conditions. We demonstrated that the MAPbI_3_ films with different initial quality can be converted into similar high-quality (large-grain and pinhole-free) MAPbI_3−*x*_Br_*x*_ films via an Ostwald-type ripening process facilitated by a simple MABr solution treatment with optimized concentration. When using a relatively high-concentration MABr solution, the I/Br ion-exchange reaction dominates and no significant Ostwald ripening is observed. It is also noteworthy that this Ostwald ripening process is absent when the MABr is replaced with MAI. The MABr selectivity could be attributed to the Br-related lower surface energy of perovskite crystallites. In addition to the significant morphological modification, the MABr-treated large-grain MAPbI_3−*x*_Br_*x*_ films exhibited improved charge-separation and/or charge-collection and passivated surface properties. Our MABr-selective Ostwald ripening not only leads to MAPbI_3−*x*_Br_*x*_ PSCs exhibiting ∼19% efficiency but also improves the device stability compared with the MAPbI_3_ PSCs. Thus, this facile and novel MABr-selective Ostwald ripening process could be used as a general and promising strategy for developing high-efficiency PSCs with improved process reproducibility and enhanced device stability.

## Methods

### Device fabrication

Fluorine-doped tin oxide (TEC15, Hartford, IN) was patterned through a wet-etching method (H_2_ evolution reaction between zinc powder and hydrochloric acid), followed by an overnight base bath-soaking (5 wt% NaOH in ethanol). A compact TiO_2_ layer was deposited by a spray pyrolysis of 0.2 M titanium diisopropoxide bis(acetylacetonate) in 1-butanol solution at 450 °C. CH_3_NH_3_PbI_3_ (or MAPbI_3_) film was fabricated on top of compact TiO_2_/fluorine-doped tin oxide using a modified solvent-engineering method. Precursor was made of 44 wt% of equimolar ratio of MAI and PbI_2_ in γ-butyrolactone (Sigma-Aldrich)/dimethyl sulfoxide (DMSO, Sigma-Aldrich; 7/3 v/v). The substrate was spun at 3,500 r.p.m. for 50 s, and a stream of toluene was injected during the spinning. Perovskite film was fully crystallized by annealing at 85 °C for 10 min. For MABr treatment, 160 μl MABr in 2-propanol solution (2–8 mg ml^−1^) was dispersed on top of the perovskite film during 4,000 r.p.m. spinning. A thermal annealing of 150 °C for 10 min was processed to remove the solvent and incorporate MABr into the perovskite film. Hole transport material was deposited on top of the perovskite film by 4,000 r.p.m. for 30 s using 2,2′,7,7′-tetrakis(N,N-dip-methoxyphenylamine)-9,9′-spirobifluorene (Spiro-OMeTAD, Merck) solution, which consists of 80 mg Spiro-OMeTAD, 30 μl bis(trifluoromethane) sulfonimide lithium salt (Li-TFSI) stock solution (500 mg Li-TFSI in 1 ml acetonitrile), 30 μl 4-tert-butylpyridine and 1 ml chlorobenzene. Finally, 150 nm of Ag film was evaporated as a counter electrode using a shadow mask.

### Characterization

The *J–V* characteristics of the cells were obtained using a 2,400 SourceMeter (Keithley) under simulated one-sun AM 1.5G illumination (100 mW cm^−2^; Oriel Sol3A Class AAA Solar Simulator, Newport Corporation). A 0.12 cm^2^ non-reflective mask was used to define the active area. The devices were stored in air (∼10–15% relative humidity) without encapsulation. Typical *J–V* scan (reverse) started from short circuit to open circuit and then back to short circuit at the rate of 100 mV s^−1^. EQE was measured using a solar cell quantum efficiency measurement system (QEX10, PV Measurements). Stabilized power output was monitored by a potentiostat (VersaSTAT MC, Princeton Applied Research) near a maximum power output point. X-ray diffraction of the perovskite thin films was performed using an X-ray diffractometer (Rigaku D/Max 2,200) with Cu Kα radiation. Absorption spectra were carried out by an UV-vis spectrometer (Cary-6000i). X-ray photoemission spectroscopy measurements were performed on a Kratos NOVA spectrometer calibrated to the Fermi edge and core-level positions of sputter-cleaned metal (Au, Ag, Cu and Mo) surfaces. XPS spectra were taken using monochromated Al Kα radiation (1,486.7 eV) at a resolution of 400 meV and fit using Pseudo-Voigt profiles. Time-resolved PL spectra were measured by exciting samples using a supercontinuum fibre laser at a wavelength of 500 nm, repetition rate of 0.1 MHz and ∼5 μW laser power with the spot size being focused down to 300 × 300 μm^2^.

### Data availability

The data supporting the findings of this study are available from the corresponding author upon request.

## Additional information

**How to cite this article:** Yang, M. *et al*. Facile fabrication of large-grain CH_3_NH_3_PbI_3−*x*_Br_*x*_ films for high-efficiency solar cells via CH_3_NH_3_Br-selective Ostwald ripening. *Nat. Commun.* 7:12305 doi: 10.1038/ncomms12305 (2016).

## Supplementary Material

Supplementary Information Supplementary Figures 1-12 and Supplementary Table 1

## Figures and Tables

**Table 1:** Photovoltaic parameters for PSCs without and with MABr treatments.

**Sample condition**	***J***_**sc**_**(mA cm**^**−2**^**)**	***V***_**oc**_ **(V)**	**FF**	**PCE** **(%)**
MAPbI_3_	19.79±0.12	1.053±0.013	0.708±0.025	14.76±0.54
MABr-treated sample	20.64±0.24	1.090±0.019	0.772±0.022	17.37±0.21

FF, fill factor; MABr, methylammonium bromide; PCE, power conversion efficiency; PSC, perovskite solar cell.

S.D.'s from 8 to 12 cells for each type of devices are given. For these devices, relatively poor-quality MAPbI_3_ films (Fig. 1a) were used as the initial films prior to MABr treatment.

**Table 2:** MABr concentration effect on the typical photovoltaic parameters.

**MABr concentration (mg ml**^**−1**^**)**	***J***_**sc**_ **(mA cm**^**−2**^**)**	***V***_**oc**_ **(V)**	**FF**	**PCE** **(%)**
0	20.54±0.07	1.052±0.027	0.724±0.012	15.64±0.63
1	21.27±0.47	1.092±0.010	0.735±0.019	17.06±0.67
2	21.86±0.12	1.120±0.010	0.754±0.020	18.50±0.39
4	21.48±0.28	1.056±0.005	0.734±0.014	16.66±0.48
8	21.27±0.16	1.049±0.011	0.665±0.031	14.83±0.47
Champion	21.60	1.120	0.793	19.12

FF, fill factor; MABr, methylammonium bromide; PCE, power conversion efficiency.

S.D.'s from 8 to 12 cells for each type of devices are given. For these devices, high-quality MAPbI_3_ films (Fig. 3a) were used as the initial films prior to MABr treatment.

## References

[b1] Kojima A., Teshima K., Shirai Y. & Miyasaka T. Organometal halide perovskites as visible-light sensitizers for photovoltaic cells. J. Am. Chem. Soc. 131, 6050–6051 (2009). 19366264 10.1021/ja809598r

[b2] Im J.-H., Lee C.-R., Lee J.-W., Park S.-W. & Park N.-G. 6.5% efficient perovskite quantum-dot-sensitized solar cell. Nanoscale 3, 4088–4093 (2011). 21897986 10.1039/c1nr10867k

[b3] Kim H.-S. . Lead iodide perovskite sensitized all-solid-state submicron thin film mesoscopic solar cell with efficiency exceeding 9%. Sci. Rep. 2, 1–7 (2012). 10.1038/srep00591 PMC3423636 22912919

[b4] Lee M. M., Teuscher J., Miyasaka T., Murakami T. N. & Snaith H. J. Efficient hybrid solar cells based on meso-superstructured organometal halide perovskites. Science 338, 643–647 (2012). 23042296 10.1126/science.1228604

[b5] Heo J. H. . Efficient inorganic-organic hybrid heterojunction solar cells containing perovskite compound and polymeric hole conductors. Nat. Photon. 7, 486–491 (2013).

[b6] Liu M., Johnston M. B. & Snaith H. J. Efficient planar heterojunction perovskite solar cells by vapour deposition. Nature 501, 395–398 (2013). 24025775 10.1038/nature12509

[b7] Li X. . Improved performance and stability of perovskite solar cells by crystal crosslinking with alkylphosphonic acid ω-ammonium chlorides. Nat. Chem. 7, 703–711 (2015). 26291941 10.1038/nchem.2324

[b8] Jeon N. J. . Solvent engineering for high-performance inorganic–organic hybrid perovskite solar cells. Nat. Mater. 13, 897–903 (2014). 24997740 10.1038/nmat4014

[b9] Im J.-H., Jang I.-H., Pellet N., Grätzel M. & Park N.-G. Growth of CH_3_NH_3_PbI_3_ cuboids with controlled size for high-efficiency perovskite solar cells. Nat. Nanotechnol. 9, 927–932 (2014). 25173829 10.1038/nnano.2014.181

[b10] Burschka J. . Sequential deposition as a route to high-performance perovskite-sensitized solar cells. Nature 499, 316–319 (2013). 23842493 10.1038/nature12340

[b11] Jeon N. J. . Compositional engineering of perovskite materials for high-performance solar cells. Nature 517, 476–480 (2015). 25561177 10.1038/nature14133

[b12] Chen W. . Efficient and stable large-area perovskite solar cells with inorganic charge extraction layers. Science 350, 944–948 (2015). 26516198 10.1126/science.aad1015

[b13] Mei A. . A hole-conductor–free, fully printable mesoscopic perovskite solar cell with high stability. Science 345, 295–298 (2014). 25035487 10.1126/science.1254763

[b14] Xiao Z. . Solvent Annealing of perovskite-induced crystal growth for photovoltaic-device efficiency enhancement. Adv. Mater. 26, 6503–6509 (2014). 25158905 10.1002/adma.201401685

[b15] Yang M. . Square-centimeter solution-processed planar CH_3_NH_3_PbI_3_ perovskite solar cells with efficiency exceeding 15%. Adv. Mater. 27, 6363–6370 (2015). 26414514 10.1002/adma.201502586

[b16] Zhao Y. & Zhu K. Efficient planar perovskite solar cells based on 1.8 eV band gap CH_3_NH_3_PbI_2_Br nanosheets via thermal decomposition. J. Am. Chem. Soc. 136, 12241–12244 (2014). 25118565 10.1021/ja5071398

[b17] Zheng Y. C. . Thermal-induced volmer–weber growth behavior for planar heterojunction perovskites solar Cells. Chem. Mater. 27, 5116–5121 (2015).

[b18] NREL, *Best Research-Cell Efficiencies* http://www.nrel.gov/ncpv/images/efficiency_chart.jpg.

[b19] Xiao M. . A fast deposition-crystallization procedure for highly efficient lead iodide perovskite thin-film solar cells. Angew. Chem. Int. Ed. 53, 9898–9903 (2014). 10.1002/anie.201405334 25047967

[b20] Ahn N. . Highly reproducible perovskite solar cells with average efficiency of 18.3% and best efficiency of 19.7% fabricated *via* Lewis base adduct of lead(II) iodide. J. Am. Chem. Soc. 137, 8696–8699 (2015). 26125203 10.1021/jacs.5b04930

[b21] Zhao Y. & Zhu K. Solution chemistry engineering toward high-efficiency perovskite solar cells. J. Phys. Chem. Lett. 5, 4175–4186 (2014). 26278951 10.1021/jz501983v

[b22] Guo Y. . Chemical pathways connecting lead(II) iodide and perovskite via polymeric plumbate(II) fiber. J. Am. Chem. Soc. 137, 15907–15914 (2015). 26617161 10.1021/jacs.5b10599

[b23] Della Gaspera E. . Ultra-thin high efficiency semitransparent perovskite solar cells. Nano Energy 13, 249–257 (2015).

[b24] Wakamiya A. . Reproducible fabrication of efficient perovskite-based solar cells: X-ray crystallographic studies on the formation of CH_3_NH_3_PbI_3_ layers. Chem. Lett. 43, 711–713 (2014).

[b25] Eperon G. E. . The importance of moisture in hybrid lead halide perovskite thin film fabrication. ACS Nano 9, 9380–9393 (2015). 26247197 10.1021/acsnano.5b03626

[b26] Wozny S. . Controlled humidity study on the formation of higher efficiency formamidinium lead triiodide-based solar cells. Chem. Mater. 27, 4814–4820 (2015).

[b27] Gao P., Grätzel M. & Nazeeruddin M. K. Organohalide lead perovskites for photovoltaic applications. Energy Environ. Sci. 7, 2448–2463 (2014).

[b28] Ratke L. & Voorhees P. W. Growth and Coarsening: Ostwald Ripening in Material Processing Springer Science & Business Media (2002).

[b29] Baldan A. Review progress in Ostwald ripening theories and their applications to nickel-base superalloys Part I: Ostwald ripening theories. J. Mater. Sci. 37, 2171–2202 (2002).

[b30] Noh J. H., Im S. H., Heo J. H., Mandal T. N. & Seok S. I. Chemical management for colorful, efficient, and stable inorganic–organic hybrid nanostructured solar cells. Nano Lett. 13, 1764–1769 (2013). 23517331 10.1021/nl400349b

[b31] Heo J.-H., Han H. J., Kim D., Ahn T. & Im S. H. 18.1% hysteresis-less inverted CH_3_NH_3_PbI_3_ planar perovskite hybrid solar cells. Energy Environ. Sci. 8, 1602–1608 (2015).

[b32] Pellet N., Teuscher J., Maier J. & Grätzel M. Transforming hybrid organic inorganic perovskites by rapid halide exchange. Chem. Mater. 27, 2181–2188 (2015).

[b33] Jang D. M. . Reversible halide exchange reaction of organometal trihalide perovskite colloidal nanocrystals for full-range band gap tuning. Nano Lett. 15, 5191–5199 (2015). 26161637 10.1021/acs.nanolett.5b01430

[b34] Harms H. A., Tetreault N., Pellet N., Bensimon M. & Grätzel M. Mesoscopic photosystems for solar light harvesting and conversion: facile and reversible transformation of metal-halide perovskites. Faraday Discuss. 176, 251–269 (2014). 25643832 10.1039/c4fd00160e

[b35] Yang S. . Formation of high-quality perovskite thin film for planar heterojunction solar cells. RSC Adv. 5, 69502–69508 (2015).

[b36] Schlipf J. . A closer look into two-step perovskite conversion with X-ray scattering. J. Phys. Chem. Lett. 6, 1265–1269 (2015). 26262985 10.1021/acs.jpclett.5b00329

[b37] Yang S. . Formation mechanism of freestanding CH_3_NH_3_PbI_3_ functional crystals: *in situ* transformation versus dissolution–crystallization. Chem. Mater. 26, 6705–6710 (2014).

[b38] Bi D. . Using a two-step deposition technique to prepare perovskite (CH_3_NH_3_PbI_3_) for thin film solar cells based on ZrO_2_ and TiO_2_ mesostructures. RSC Adv. 3, 18762–18766 (2013).

