# Supplementary material for: Facile fabrication of large-grain CH3NH3PbI3−xBrx films for high-efficiency solar cells via CH3NH3Br-selective Ostwald ripening
Source: Nat Commun. 2016 Aug 1;7:12305. doi: 10.1038/ncomms12305 (PMC4974626; doi:10.1038/ncomms12305)
Supplement: Supplementary Information — Supplementary Figures 1-12 and Supplementary Table 1 [file ncomms12305-s1.pdf]

Supplementary Information

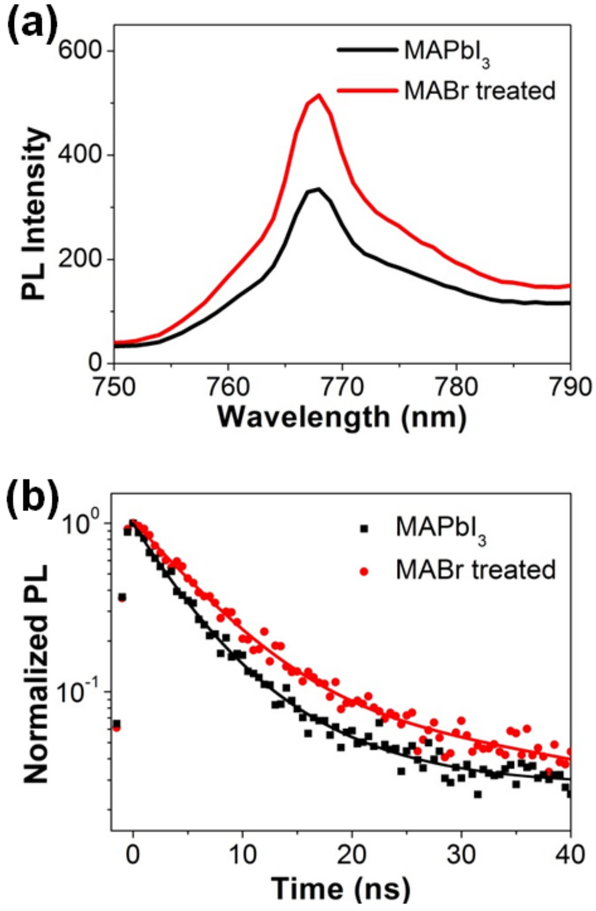

Supplementary Figure 1. Impact of MABr treatment on the photoluminescence (PL) properties of $MAPbI_3$ thin film. (**a**) Steady-state PL and (**b**) time-resolved PL spectra of $MAPbI_3$ films with and without MABr treatment. These transients follow bi-exponential decay. The weighted average of lifetime increases approximately from about 5 ns for $MAPbI_3$ to 9 ns for MABr-treated perovskite film.

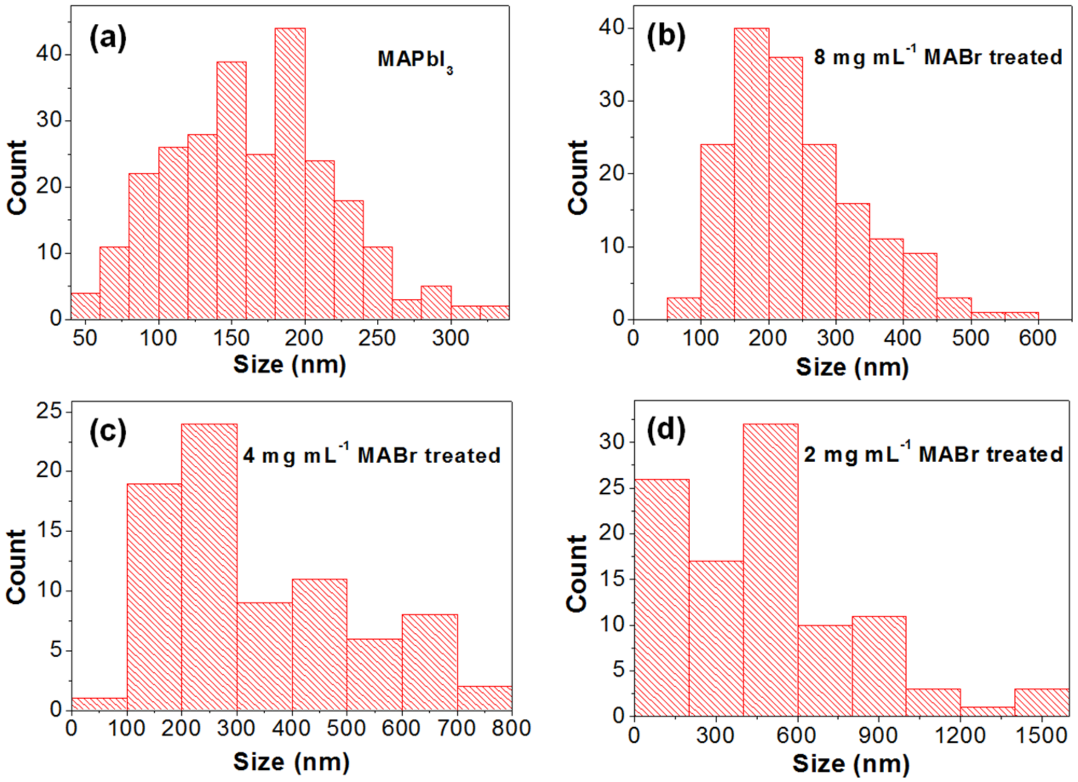

Supplementary Figure 2. Grain-size distribution. Perovskite films (**a**) without and with (**b**) 8, (**c**) 4, and (**d**) 2 mg mL^{-1} MABr solution treatment.

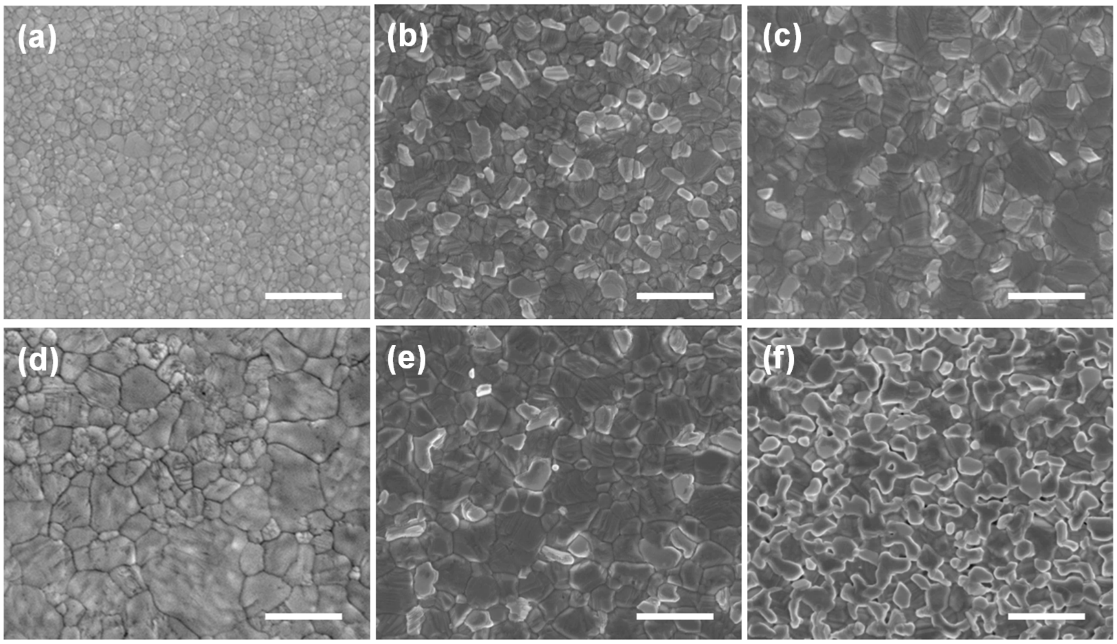

Supplementary Figure 3. Top-view SEM images of perovskite thin films. (**a**) As-prepared $MAPbI_3$ thin films and those treated with (**b**) IPA solvent and (**c**) 1 mg mL^{-1} MABr, (**d**) 2 mg mL^{-1} MABr, (**e**) 2.8 mg mL^{-1} MAI, and (**f**) 1.2 mg mL^{-1} MACl solutions, respectively. Scale bars, 1 μm.

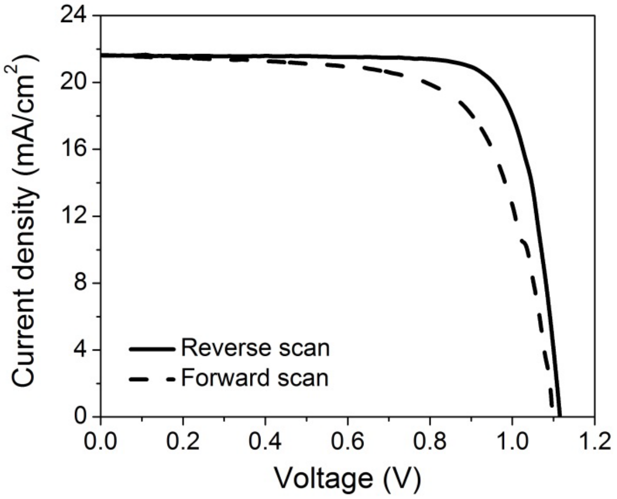

Supplementary Figure 4. Current density-voltage (*J–V*) curves with both forward and reverse scans for the "champion" cell. For the reverse scan, the device shows a power conversion efficiency (*PCE*) of 19.12%, with short-circuit current density (J_{sc}) of 21.60 mA/cm^2, open-circuit voltage (V_{oc}) of 1.12 V, and fill factor (*FF*) of 0.793. For the forward scan, the device shows a *PCE* of 16.43%, with J_{sc} of 21.61 mA/cm^2, V_{oc} of 1.097 V, and *FF* of 0.693.

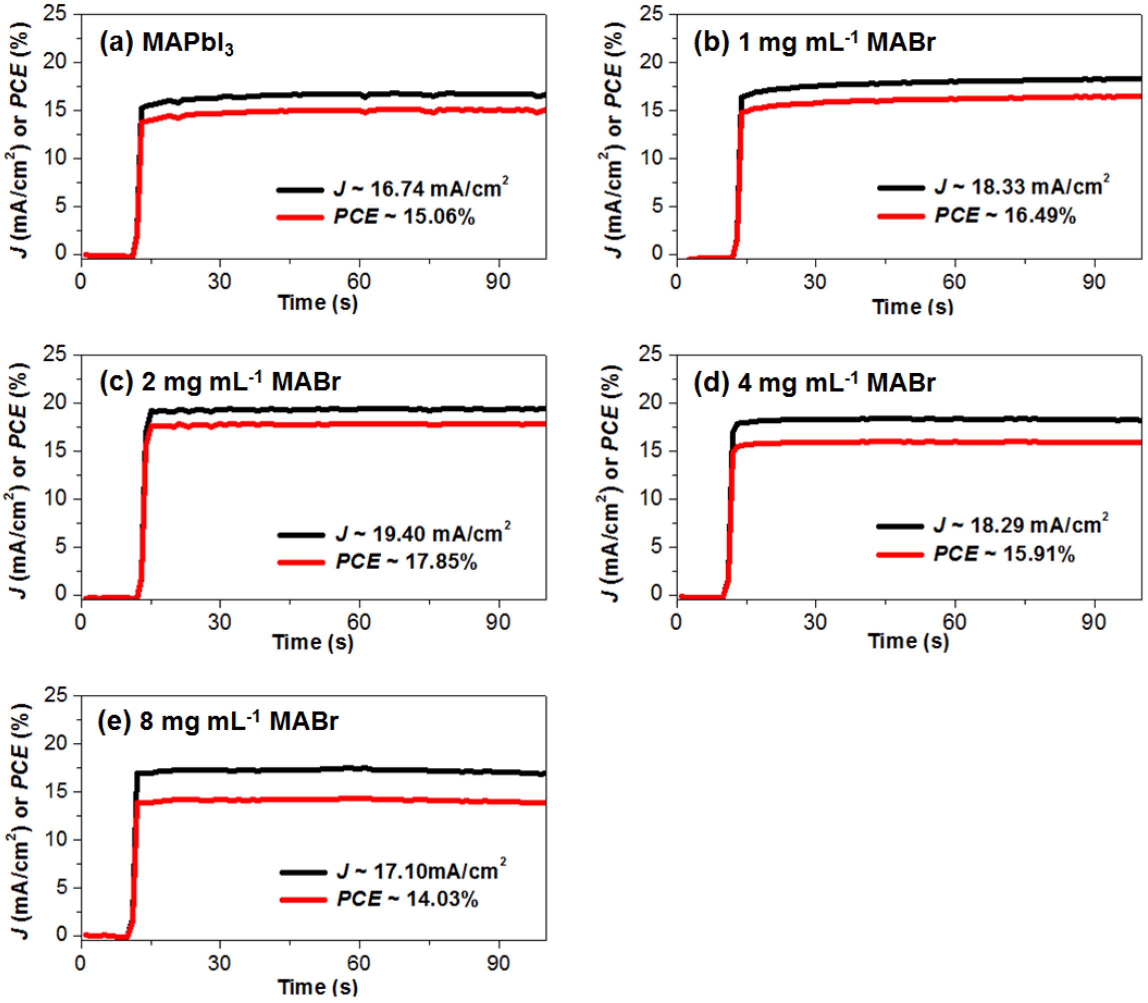

Supplementary Figure 5. MABr concentration effect on the stabilized photocurrent density and power conversion efficiency biased near the maximum power point. Perovskite thin films are (**a**) $MAPbI_3$ and those treated with (**b**) 1, (**c**) 2, (**d**) 4, and (**e**) 8 mg mL^{-1} MABr solutions.

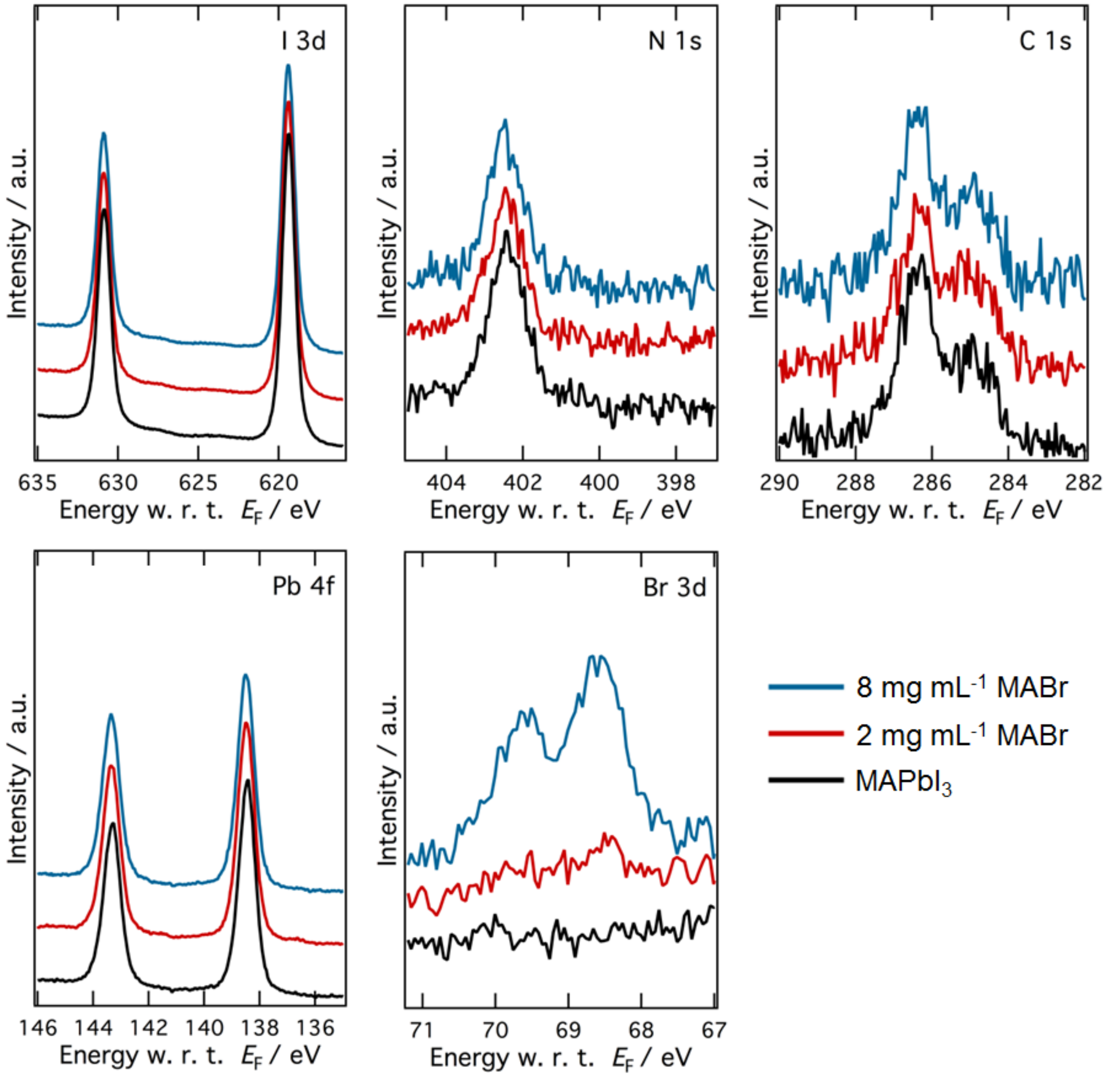

Supplementary Figure 6. X-ray photoelectron spectroscopy (XPS) core-level spectra. Plots of XPS spectra of I 3d, N 1s, C 1s, Pb 4f, and Br 3d levels for $MAPbI_3$ thin films and those treated with low (2 mg mL^{-1}) and high (8 mg mL^{-1}) concentration MABr solutions. Despite an increase in the bromine content with increased concentration of the MABr solution, no further changes is observed in film stoichiometry upon MABr post-treatment. Moreover, the absence of core-level shifts indicates that no significant change in the Fermi level position occurs with Ostwald ripening.

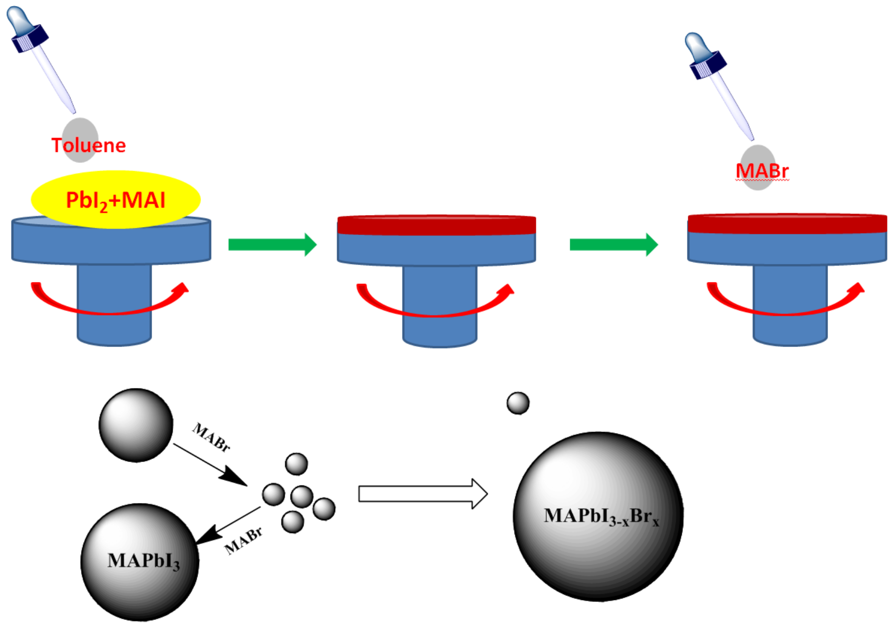

Supplementary Figure 7. Schematic illustration. Procedure and mechanism for the MABr selective Ostwald ripening process for the perovskite crystal growth.

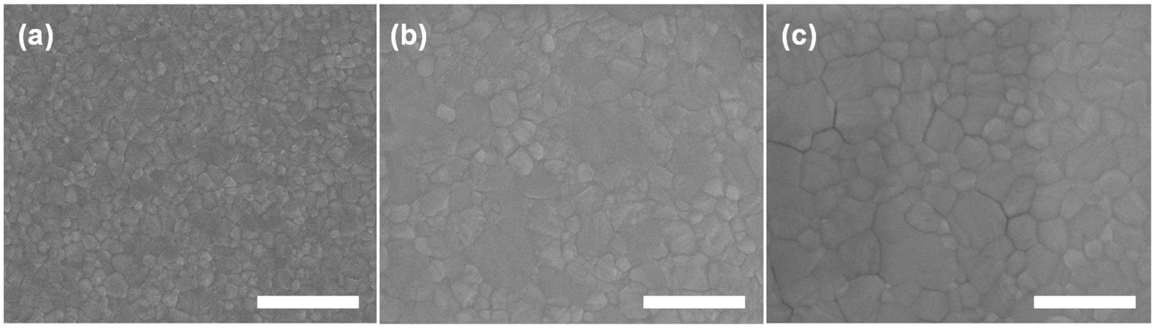

Supplementary Figure 8. Top-view SEM images of perovskite thin films. (**a**) As-prepared $MAPbI_3$ and those treated with 2 mg mL^{-1} MABr solution (**b**) before and (**c**) after annealing treatment. Scale bars, 1 μm.

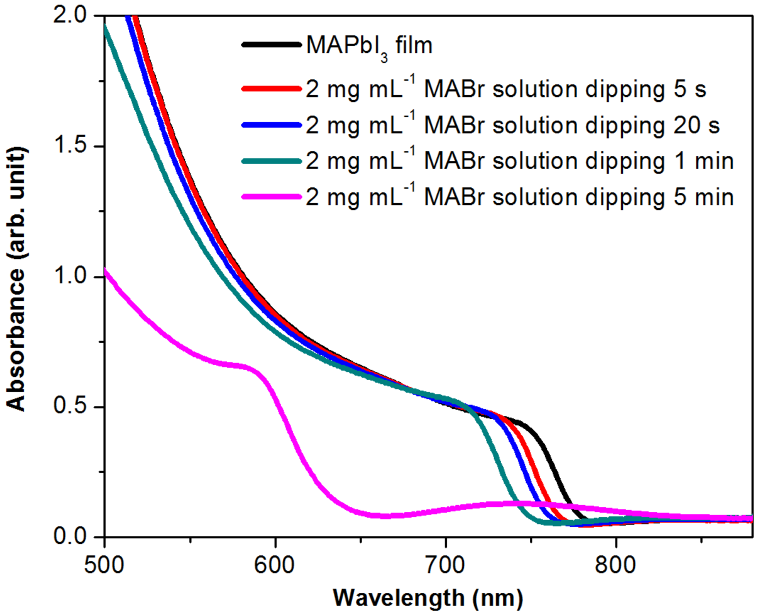

Supplementary Figure 9. Ultraviolet-visible absorption spectra. Perovskite thin films were subjected to dipping treatment in 2 mg mL^{-1} MABr solution for various durations.

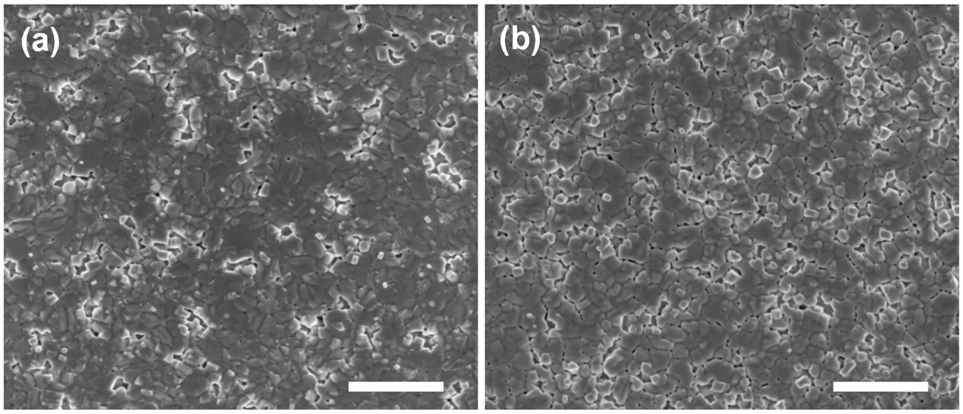

Supplementary Figure 10. Top-view scanning electron microscopy images of perovskite thin films. $MAPbI_3$ thin films were dipped in 2 mg mL^{-1} MABr solution for (**a**) 20 s and (**b**) 5 min. Scale bars, 1 μm.

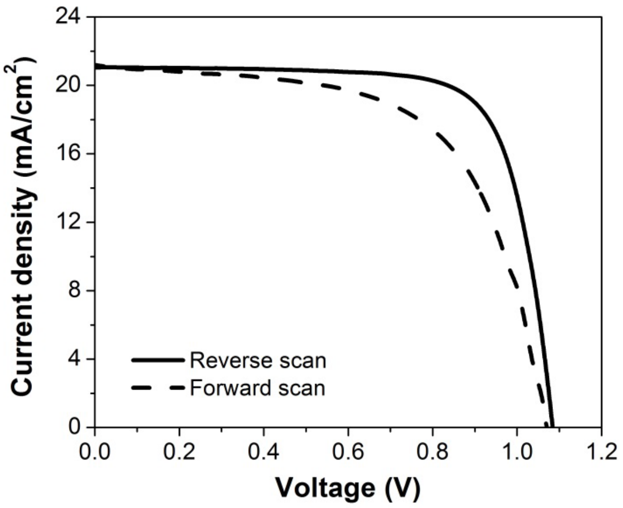

Supplementary Figure 11. J–V curves with both forward and reverse scans for the "champion" cell with MAI treatment. For the reverse scan, the device shows a *PCE* of 17.11%, with J_{sc} of 21.05 mA/cm^2, V_{oc} of 1.084 V, and *FF* of 0.749. For the forward scan, the device shows a *PCE* of 13.93%, with J_{sc} of 21.18 mA/cm^2, V_{oc} of 1.071 V, and *FF* of 0.614.

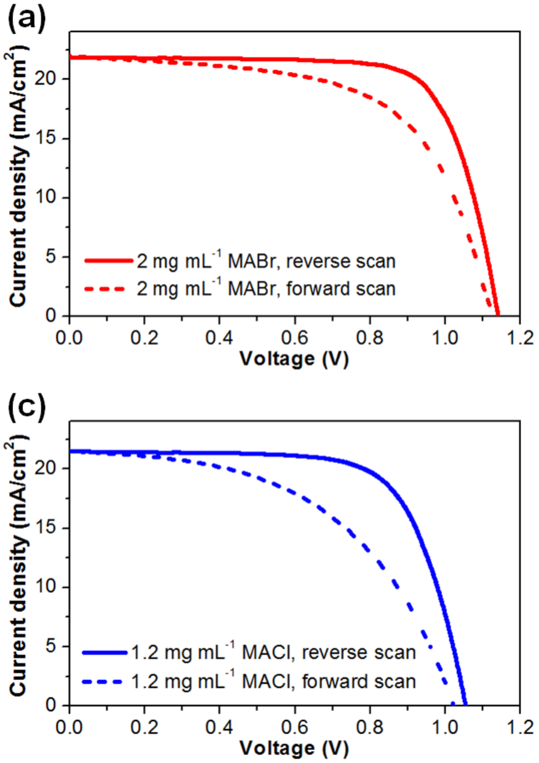

Supplementary Figure 12. *J*–*V* curves with both forward and reverse scans for PSCs with various treatments. The perovskite films were treated with (**a**) 2 mg mL^{-1} MABr, (**b**) 2.8 mg mL^{-1} MAI, (**c**) 1.2 mg mL^{-1} MACl solutions, and (**d**) IPA solvent, respectively.

Supplementary Table 1. Photovoltaic parameters for perovskite solar cells with various treatments. Standard deviations from 8–12 cells for each type of devices are given.

Treatment solution	J_{sc} (mA cm^{-2})	V_{oc} (V)	*FF* (%)	*PCE* (%)
2 mg mL^{-1} MABr	21.86 ± 0.12	1.120 ± 0.010	0.754 ± 0.020	18.50 ± 0.39
2.8 mg mL^{-1} MAI	21.35 ± 0.19	1.071 ± 0.006	0.748 ± 0.006	17.11 ± 0.21
1.2 mg mL^{-1} MACl	21.52 ± 0.14	1.061 ± 0.009	0.696 ± 0.017	15.89 ± 0.41
IPA	20.62 ± 0.43	1.067 ± 0.009	0.728 ± 0.031	16.03 ± 1.08
